# Supra­molecular inter­actions in the 1:2 co-crystal of 4,4′-bipyridine and 3-chloro­thio­phene-2-carb­oxy­lic acid

**DOI:** 10.1107/S2056989016013724

**Published:** 2016-09-05

**Authors:** Olakkandiyil Prajina, Packianathan Thomas Muthiah, David K. Geiger

**Affiliations:** a School of Chemistry, Bharathidasan University, Tiruchirappalli 620 024, Tamilnadu, India; b Department of Chemistry, SUNY-College at Geneseo, Geneseo, New York 14454, USA

**Keywords:** crystal structure, 3-chloro­thio­phene-2-carb­oxy­lic acid, 4,4′-bi­pyridine, O—H⋯N-based synthon, co-crystal

## Abstract

The asymmetric unit of the title compound, 2C_5_H_3_ClO_2_S·C_10_H_8_N_2_, is comprised of a mol­ecule of 3-chloro­thio­phene-2-carb­oxy­lic acid (3TPC) and half of a mol­ecule of 4,4′-bi­pyridine (BPY). A distinctive O—H⋯N-based synthon is present. Cl⋯Cl and π–π stacking inter­actions further stabilize the crystal structure, forming a two-dimensional network parallel to the *bc* plane.

## Abstract 2

The asymmetric unit comprises of one 3-chloro­thio­phene-2-carb­oxy­lic acid (3TPC) and one half of a 4,4′-bi­pyridine (BPY) mol­ecule linked together *via* an O–H⋯N hydrogen bond.

## Chemical context   

Structurally homogeneous crystalline solids in well defined stochiometry are called co-crystals. In recent years, the physicochemical properties of active pharmaceutical ingredients have been improved widely with the use of co-crystals (Lemmerer & Bernstein, 2010 ▸). Supra­molecular synthons – modular representation of primary recognition between functional groups – are of great importance in providing an effective strategy for designing solids in crystal engineering. All geometrical and chemical information of mol­ecular recognition is contained in the structural units called synthons. In the context of co-crystal formation, heterosynthons provide a predictive justification in terms of unique inter­molecular inter­actions (Mukherjee *et al.*, 2011 ▸, 2013 ▸). There are many literature cases of O—H⋯N-bonded inter­actions between acid and pyridine-based systems (Shattock *et al.*, 2008 ▸; Lemmerer *et al.*, 2015 ▸). 4,4′-Bi­pyridine (BPY) is a weak bidentate base commonly used in crystal engineering on account of its bridging abilities. It also acts as the co-crystal former in the present study because it readily participates in hydrogen bonds with carboxyl-attached organic mol­ecules (Pan *et al.*, 2008 ▸).
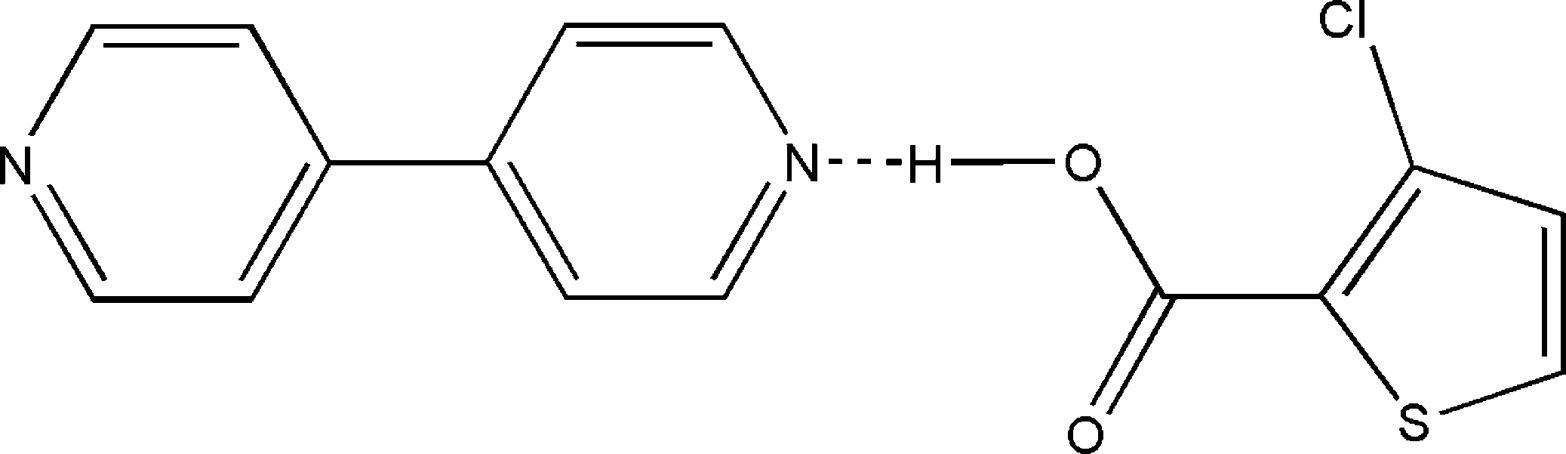


Inter­molecular inter­actions involving halogen substituents, particularly chlorides, play an important role in mol­ecular self-assembly in supra- and biomolecular systems to prepare highly stereoregular organic polymers. It has been observed that these inter­actions act as a tool in crystal engineering to enhance crystal formation and for the design of supra­molecular aggregates (Cavallo *et al.*, 2016 ▸). In this context, the study of the effect of various halogens on the mol­ecular packing and crystalline architecture of solids has attracted great attention (Csöregh *et al.*, 2001 ▸). The structure-forming ability of Cl⋯Cl inter­actions in assembling chains, ladders, two-dimensional sheets, *etc*. has been studied extensively (Navon *et al.*, 1997 ▸; Metrangolo & Resnati, 2014 ▸). It is based on the values of the two C—Hal⋯Hal angles, θ1 and θ2 (Vener *et al.*, 2013 ▸).

## Structural commentary   

The asymmetric unit of the title compound (I) consists of a mol­ecule of 3-chloro­thio­phene-2-carb­oxy­lic acid, 3TPC, and a half of a mol­ecule of 4,4′-bi­pyridine, BPY, which is located on a crystallographic inversion center. The inter­nal angle at N1 in BPY is 117.1 (3)° and bond lengths [N1-C6= 1.336 (5) Å and N1-C10 = 1.329 (5) Å] agree with those reported for neutral BPY structures (see for example Jennifer & Mu­thiah, 2014 ▸; Atria *et al.*, 2014 ▸; Moon & Park, 2012 ▸; Qin, 2011 ▸; Najafpour *et al.*, 2008 ▸). The two external bond angles at the carbon of the carboxyl group are 123.7 (3)° and 112.4 (3)°. The high discrepancy between these two angles is typical of an unionized carboxyl group, as are the C=O distance of 1.219 (4) Å and C—OH distance of 1.3254 (5) Å (see for example Prajina *et al.*, 2016 ▸; Atria *et al.*, 2014 ▸; Jennifer & Mu­thiah, 2014 ▸; Qin, 2011 ▸). The bond distances and angles of the thio­phene ring agree with those in structures reported earlier (Zhang *et al.*, 2014 ▸).

## Supra­molecular features   

**Figure 1:**
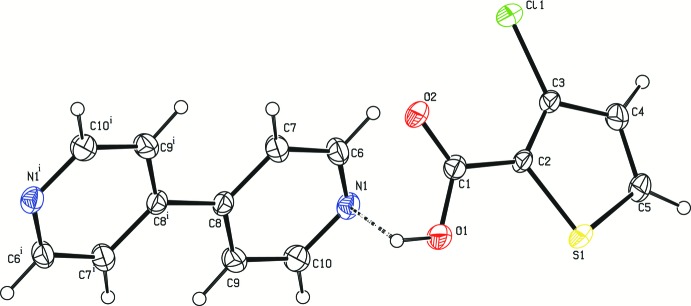
The asymmetric unit of the title compound, showing the atom-numbering scheme. Displacement ellipsoids are drawn at the 30% probability level. The dashed line represents the O—H⋯N hydrogen bond. [Symmetry code: (i) −*x* + 1, −*y* + 1, −*z* + 1.]

**Figure 2:**
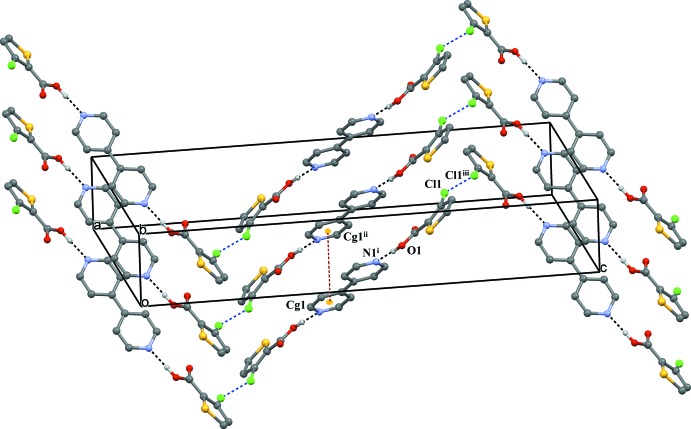
A view of the O—H⋯N hydrogen bonds (black dashed lines), π–π stacking (brown dashed lines) and Cl⋯Cl inter­actions (blue dashed lines). Symmetry codes: (i) *x*, *y* − 1, *z;* (ii) 1 − *x*, 2 − *y*, 1 – *z*; (iii) 1 − *x*, *y*, 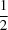 − *z*.

3TPC and BPY are inter­connected *via* O—H⋯N hydrogen-bonding inter­actions between (O1—H1) of the carboxyl group and the nitro­gen (N1) of BPY (Table 1 ▸ and Fig. 1 ▸). This O—H⋯N hydrogen bond is a frequently observed supra­molecular synthon in crystal engineering involving a carb­oxy­lic acid and a pyridine system (Dubey & Desiraju, 2015 ▸; Lemmerer & Bernstein, 2010 ▸; Mukherjee *et al.*, 2011 ▸, 2013 ▸; Prajina *et al.*, 2016 ▸; Seaton, 2014 ▸; Thomas *et al.*, 2010 ▸). This supra­molecular synthon is also present in the co-crystal of 5-chloro­thio­phene-2-carb­oxy­lic acid with BPY (5TPC44BIPY) and in the co-crystal of thio­phene-2-carb­oxy­lic acid with BPY reported from our laboratory (Jennifer & Mu­thiah, 2014 ▸). The co-crystal 5TPC44BIPY and the title co-crystal differ only in the position of chlorine in the thio­phene ring with the same base. A chloro derivative was chosen as co-mol­ecule with the expectation that the presence of a Cl atom would result in halogen–halogen inter­actions. As expected, a Cl⋯Cl inter­action plays the key role in connecting the O—H⋯N hydrogen-bonded units to form an infinite zigzag chain, *i.e*., the three-mol­ecule aggregates are further linked to similar neighbouring aggregates through Cl⋯Cl inter­actions [3.3925 (12) Å, C3—Cl1⋯Cl1^iii^ = 151.71 (1)°; symmetry code: (iii) 1 − *x*, *y*, 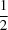 − *z*] (Vener *et al.*, 2013 ▸; Sarma & Desiraju, 1986 ▸; Capdevila-Cortada *et al.*, 2014 ▸). The hydrogen-bonded units are stabilized *via* π–π stacking inter­actions between the aromatic systems of BPY mol­ecules [*Cg*1⋯*Cg*1^ii^ = 3.794 (2) Å; *Cg*1 is the centroid of the N1/C6/C7/C8/C9/C10 ring; symmetry code: (ii) 1 − *x*, 2 − *y*, 1 − *z*]. The perpendicular distance between two parallel mol­ecules is 3.4812 (15) Å. This weak inter­action holds the hydrogen-bonded chains together, supporting a two-dimensional supra­molecular network parallel to the *bc* plane, as seen in Fig. 2 ▸.

## Database survey   

In the title compound, the most dominant inter­action is the O—H⋯N hydrogen bond formed between a carboxyl group and a pyridine N atom (Fig. 1 ▸). The length of this hydrogen bond [O⋯N = 2.659 (4) Å] is very close to those of O—H⋯N bonds found in similar reported co-crystals, such as in the adduct of 2,5-dihy­droxy-1,4-benzo­quinone and BPY (Cowan *et al.*, 2001 ▸) and in the co-crystal of BPY with *N*,*N*′-dioxide-3-hy­droxy-2-naphthoic acid (1/2) (Lou & Huang, 2007 ▸) and in a series of nine co-crystals involving acridine and benzoic acids (Kowalska *et al.*, 2015 ▸). The angle of the hydrogen bond formed between the 3CTPC and BPY mol­ecules is 178 (5)°. A similar value is found in the co-crystal of BPY with 3,5-di­nitro benzoic acid for which the O⋯N distance is 2.547 (2) Å (Thomas *et al.*, 2010 ▸). In the crystal structure of the co-crystal of adamantane-1,3-di­carb­oxy­lic acid and 4,4′-bi­pyridine, π–π inter­actions connect the O—H⋯N hydrogen-bonded zigzag chains, supporting a two-dimensional network (Pan *et al.*, 2008 ▸).

## Synthesis and crystallization   

To 10 ml of a hot methanol solution of 3TPC (40.6 mg, 25 mmol), 10 ml of a hot methano­lic solution of BPY (39.0 mg, 25 mmol) was added. The resulting solution was warmed over a water bath for half an hour and then kept at room temperature for crystallization. After a week, clear yellow plates were obtained. The crystal used for X-ray diffraction data collection was cut from a larger crystal.

## Refinement   

Crystal data, data collection and structure refinement details are summarized in Table 2 ▸. All hydrogen atoms were located in difference Fourier maps. The hydrogen atoms bonded to carbon were refined using a riding model with C—H = 0.95 Å and *U*_iso_(H) = 1.2*U*_eq_(C). The carb­oxy­lic acid hydrogen atom was freely refined, including its isotropic displacement parameter.

## Supplementary Material

Crystal structure: contains datablock(s) I. DOI: 10.1107/S2056989016013724/hg5476sup1.cif

Structure factors: contains datablock(s) I. DOI: 10.1107/S2056989016013724/hg5476Isup2.hkl

Supporting information file. DOI: 10.1107/S2056989016013724/hg5476Isup3.cml

CCDC reference: 1501060

Additional supporting information: crystallographic information; 3D view; checkCIF report

## Figures and Tables

**Table 1:** Hydrogen-bond geometry (Å, °)

*D*—H⋯*A*	*D*—H	H⋯*A*	*D*⋯*A*	*D*—H⋯*A*
O1—H1⋯N1^i^	0.89 (5)	1.77 (5)	2.659 (4)	178 (5)

Symmetry code: (i) 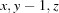.

**Table 2:** Experimental details

Crystal data	
Chemical formula	2C_5_H_3_ClO_2_S·C_10_H_8_N_2_
*M*_r_	481.35
Crystal system, space group	Monoclinic, *C*2/*c*
Temperature (K)	200
*a*, *b*, *c* (Å)	13.538 (4), 5.1230 (18), 30.167 (10)
β (°)	95.968 (9)
*V* (Å^3^)	2080.8 (12)
*Z*	4
Radiation type	Mo *K*α
μ (mm^−1^)	0.54
Crystal size (mm)	0.50 × 0.50 × 0.10

Data collection	
Diffractometer	Bruker *SMART* X2S benchtop
Absorption correction	Multi-scan (*SADABS*; Bruker, 2013 ▸)
*T*_min_, *T*_max_	0.69, 0.95
No. of measured, independent and observed [*I* > 2σ(*I*)] reflections	10427, 1907, 1563
*R*_int_	0.078
(sin θ/λ)_max_ (Å^−1^)	0.607

Refinement	
*R*[*F* ^2^ > 2σ(*F* ^2^)], *wR*(*F* ^2^), *S*	0.055, 0.136, 1.19
No. of reflections	1907
No. of parameters	140
H-atom treatment	H atoms treated by a mixture of independent and constrained refinement
Δρ_max_, Δρ_min_ (e Å^−3^)	0.37, −0.32

Computer programs: *APEX2* (Bruker, 2013 ▸), *SAINT* (Bruker, 2013 ▸), *SHELXS97* (Sheldrick, 2008 ▸), *SHELXL2014* (Sheldrick, 2015 ▸), *PLATON* (Spek, 2009 ▸), *Mercury* (Macrae *et al.*, 2008 ▸) and *publCIF* (Westrip, 2010 ▸).

## supplementary crystallographic information

### Crystal data

**Table:**

2C_5_H_3_ClO_2_S·C_10_H_8_N_2_	*F*(000) = 984
*M**_r_* = 481.35	*D*_x_ = 1.537 Mg m^−^^3^
Monoclinic, *C*2/*c*	Mo *K*α radiation, λ = 0.71073 Å
*a* = 13.538 (4) Å	Cell parameters from 3438 reflections
*b* = 5.1230 (18) Å	θ = 2.7–24.8°
*c* = 30.167 (10) Å	µ = 0.54 mm^−^^1^
β = 95.968 (9)°	*T* = 200 K
*V* = 2080.8 (12) Å^3^	Plate, clear colourless
*Z* = 4	0.50 × 0.50 × 0.10 mm

### Data collection

**Table:**

Bruker SMART X2S benchtop diffractometer	1563 reflections with *I* > 2σ(*I*)
Radiation source: sealed microfocus tube	*R*_int_ = 0.078
ω scans	θ_max_ = 25.6°, θ_min_ = 1.4°
Absorption correction: multi-scan (SADABS; Bruker, 2013)	*h* = −16→16
*T*_min_ = 0.69, *T*_max_ = 0.95	*k* = −6→6
10427 measured reflections	*l* = −36→36
1907 independent reflections	

### Refinement

**Table:**

Refinement on *F*^2^	0 restraints
Least-squares matrix: full	Hydrogen site location: mixed
*R*[*F*^2^ > 2σ(*F*^2^)] = 0.055	H atoms treated by a mixture of independent and constrained refinement
*wR*(*F*^2^) = 0.136	*w* = 1/[σ^2^(*F*_o_^2^) + (0.0431*P*)^2^ + 4.3057*P*] where *P* = (*F*_o_^2^ + 2*F*_c_^2^)/3
*S* = 1.19	(Δ/σ)_max_ < 0.001
1907 reflections	Δρ_max_ = 0.37 e Å^−^^3^
140 parameters	Δρ_min_ = −0.32 e Å^−^^3^

### Special details

**Table:**

Geometry. All esds (except the esd in the dihedral angle between two l.s. planes) are estimated using the full covariance matrix. The cell esds are taken into account individually in the estimation of esds in distances, angles and torsion angles; correlations between esds in cell parameters are only used when they are defined by crystal symmetry. An approximate (isotropic) treatment of cell esds is used for estimating esds involving l.s. planes.

### Fractional atomic coordinates and isotropic or equivalent isotropic displacement parameters (Å^2^)

**Table:**

	*x*	*y*	*z*	*U*_iso_*/*U*_eq_	
N1	0.3544 (2)	0.9782 (6)	0.54889 (10)	0.0363 (7)	
C8	0.4696 (2)	0.6000 (7)	0.51024 (11)	0.0312 (8)	
C7	0.4776 (3)	0.6415 (8)	0.55606 (12)	0.0406 (9)	
H7	0.5227	0.5401	0.5752	0.049*	
C6	0.4200 (3)	0.8304 (8)	0.57370 (11)	0.0385 (9)	
H6	0.4275	0.8563	0.6051	0.046*	
C10	0.3468 (3)	0.9421 (8)	0.50506 (12)	0.0423 (9)	
H10	0.3016	1.0481	0.4868	0.051*	
C9	0.4017 (3)	0.7571 (7)	0.48451 (12)	0.0386 (9)	
H9	0.3931	0.7377	0.4530	0.046*	
S1	0.12214 (6)	0.6112 (2)	0.64475 (3)	0.0420 (3)	
Cl1	0.40721 (6)	0.8001 (2)	0.70764 (3)	0.0447 (3)	
O1	0.24836 (19)	0.2918 (6)	0.59585 (9)	0.0462 (7)	
O2	0.39160 (18)	0.3397 (5)	0.63983 (8)	0.0411 (6)	
C2	0.2498 (2)	0.5841 (7)	0.65494 (11)	0.0304 (7)	
C5	0.1177 (3)	0.8526 (8)	0.68316 (12)	0.0454 (10)	
H5	0.0579	0.9341	0.6899	0.054*	
C4	0.2091 (3)	0.9160 (8)	0.70321 (12)	0.0414 (9)	
H4	0.2210	1.0482	0.7252	0.050*	
C3	0.2846 (2)	0.7602 (7)	0.68722 (10)	0.0312 (8)	
C1	0.3043 (3)	0.3936 (7)	0.63023 (11)	0.0325 (8)	
H1	0.283 (4)	0.184 (10)	0.5800 (16)	0.072 (15)*	

### Atomic displacement parameters (Å^2^)

**Table:**

	*U*^11^	*U*^22^	*U*^33^	*U*^12^	*U*^13^	*U*^23^
N1	0.0315 (16)	0.0350 (17)	0.0435 (17)	−0.0014 (13)	0.0097 (13)	−0.0083 (13)
C8	0.0258 (18)	0.0349 (19)	0.0340 (17)	−0.0042 (15)	0.0083 (14)	−0.0031 (14)
C7	0.041 (2)	0.049 (2)	0.0321 (18)	0.0065 (18)	0.0083 (16)	−0.0011 (16)
C6	0.042 (2)	0.042 (2)	0.0330 (18)	−0.0001 (17)	0.0108 (15)	−0.0080 (15)
C10	0.040 (2)	0.046 (2)	0.041 (2)	0.0100 (18)	0.0024 (16)	−0.0040 (17)
C9	0.041 (2)	0.042 (2)	0.0331 (18)	0.0043 (17)	0.0029 (15)	−0.0075 (15)
S1	0.0207 (5)	0.0602 (7)	0.0448 (5)	0.0069 (4)	0.0021 (4)	−0.0076 (4)
Cl1	0.0261 (5)	0.0630 (7)	0.0440 (5)	0.0012 (4)	−0.0013 (4)	−0.0081 (4)
O1	0.0313 (14)	0.0611 (19)	0.0459 (15)	0.0083 (13)	0.0023 (12)	−0.0207 (14)
O2	0.0254 (13)	0.0515 (17)	0.0466 (14)	0.0130 (12)	0.0047 (11)	−0.0053 (12)
C2	0.0196 (16)	0.040 (2)	0.0316 (17)	0.0064 (14)	0.0048 (13)	0.0036 (14)
C5	0.031 (2)	0.061 (3)	0.045 (2)	0.0188 (19)	0.0092 (16)	−0.0057 (18)
C4	0.038 (2)	0.051 (2)	0.0357 (19)	0.0114 (18)	0.0063 (16)	−0.0071 (16)
C3	0.0233 (17)	0.042 (2)	0.0282 (16)	0.0065 (15)	0.0034 (13)	0.0029 (14)
C1	0.0294 (19)	0.0352 (19)	0.0335 (17)	0.0025 (15)	0.0056 (14)	0.0019 (14)

### Geometric parameters (Å, º)

**Table:**

N1—C10	1.329 (5)	S1—C2	1.729 (3)
N1—C6	1.336 (5)	Cl1—C3	1.721 (3)
C8—C7	1.391 (5)	O1—C1	1.325 (4)
C8—C9	1.395 (5)	O1—H1	0.89 (5)
C8—C8^i^	1.489 (7)	O2—C1	1.219 (4)
C7—C6	1.384 (5)	C2—C3	1.374 (5)
C7—H7	0.9500	C2—C1	1.472 (5)
C6—H6	0.9500	C5—C4	1.359 (6)
C10—C9	1.389 (5)	C5—H5	0.9500
C10—H10	0.9500	C4—C3	1.421 (5)
C9—H9	0.9500	C4—H4	0.9500
S1—C5	1.700 (4)		
			
C10—N1—C6	117.1 (3)	C1—O1—H1	112 (3)
C7—C8—C9	116.3 (3)	C3—C2—C1	129.8 (3)
C7—C8—C8^i^	121.8 (4)	C3—C2—S1	109.7 (3)
C9—C8—C8^i^	121.8 (4)	C1—C2—S1	120.5 (3)
C6—C7—C8	120.0 (3)	C4—C5—S1	112.4 (3)
C6—C7—H7	120.0	C4—C5—H5	123.8
C8—C7—H7	120.0	S1—C5—H5	123.8
N1—C6—C7	123.4 (3)	C5—C4—C3	111.7 (3)
N1—C6—H6	118.3	C5—C4—H4	124.2
C7—C6—H6	118.3	C3—C4—H4	124.2
N1—C10—C9	123.4 (3)	C2—C3—C4	113.8 (3)
N1—C10—H10	118.3	C2—C3—Cl1	125.4 (3)
C9—C10—H10	118.3	C4—C3—Cl1	120.9 (3)
C10—C9—C8	119.8 (3)	O2—C1—O1	123.9 (3)
C10—C9—H9	120.1	O2—C1—C2	123.7 (3)
C8—C9—H9	120.1	O1—C1—C2	112.4 (3)
C5—S1—C2	92.46 (18)		
			
C9—C8—C7—C6	0.0 (5)	S1—C5—C4—C3	−0.9 (5)
C8^i^—C8—C7—C6	179.7 (4)	C1—C2—C3—C4	179.3 (3)
C10—N1—C6—C7	−1.4 (6)	S1—C2—C3—C4	−0.4 (4)
C8—C7—C6—N1	0.8 (6)	C1—C2—C3—Cl1	−0.3 (6)
C6—N1—C10—C9	1.4 (6)	S1—C2—C3—Cl1	180.0 (2)
N1—C10—C9—C8	−0.7 (6)	C5—C4—C3—C2	0.9 (5)
C7—C8—C9—C10	−0.1 (5)	C5—C4—C3—Cl1	−179.5 (3)
C8^i^—C8—C9—C10	−179.7 (4)	C3—C2—C1—O2	10.6 (6)
C5—S1—C2—C3	−0.1 (3)	S1—C2—C1—O2	−169.7 (3)
C5—S1—C2—C1	−179.8 (3)	C3—C2—C1—O1	−168.0 (4)
C2—S1—C5—C4	0.6 (3)	S1—C2—C1—O1	11.7 (4)

Symmetry code: (i) −*x*+1, −*y*+1, −*z*+1.

### Hydrogen-bond geometry (Å, º)

**Table:**

*D*—H···*A*	*D*—H	H···*A*	*D*···*A*	*D*—H···*A*
O1—H1···N1^ii^	0.89 (5)	1.77 (5)	2.659 (4)	178 (5)

Symmetry code: (ii) *x*, *y*−1, *z*.
